# Activation of Canine, Mouse and Human TLR2 and TLR4 by Inactivated *Leptospira* Vaccine Strains

**DOI:** 10.3389/fimmu.2022.823058

**Published:** 2022-03-21

**Authors:** Andreja Novak, Elder Pupo, Esther van’t Veld, Victor P. M. G. Rutten, Femke Broere, Arjen Sloots

**Affiliations:** ^1^ Division Infectious Diseases and Immunology, Department Biomolecular Health Sciences, Faculty of Veterinary Medicine, Utrecht University, Utrecht, Netherlands; ^2^ Department of Product Characterization and Formulation, Intravacc, Bilthoven, Netherlands; ^3^ Center for Cell Imaging (CCI), Division Cell Biology, Metabolism and Cancer, Department Biomolecular Health Sciences, Faculty of Veterinary Medicine, Utrecht University, Utrecht, Netherlands; ^4^ Department of Veterinary Tropical Diseases, Faculty of Veterinary Science, University of Pretoria, Pretoria, South Africa; ^5^ Division Internal Medicine of Companion Animals, Department Clinical Science, Faculty of Veterinary Medicine, Utrecht University, Utrecht, Netherlands

**Keywords:** *Leptospira*, toll-like receptor, monocyte-derived dendritic cells, innate immunity, vaccine, canine

## Abstract

Canine *Leptospira* vaccines contain inactivated strains of pathogenic *Leptospira*, the causative agents of leptospirosis. For an effective response to vaccination, activation of the innate immune system *via* pattern recognition receptors such as TLRs is crucial. However, it is not known which TLRs are activated by *Leptospira* in dogs. To investigate the involvement of canine TLR2, TLR4, and TLR5 in the recognition of *Leptospira*, we stimulated canine moDC and reporter cells expressing canine TLR2 with either whole-inactivated bacteria or purified LPS of *Leptospira* strains, representing the serogroups generally used in canine leptospirosis vaccines. Using the endotoxin neutralizing reagent polymyxin B and TLR4 antagonist RS-LPS, we demonstrate that *Leptospira* LPS and canine TLR4 are involved in IL-1β production as well as in the uptake of inactivated *Leptospira* in canine moDC. Furthermore, polymyxin B only partially inhibited IL-1β production induced by inactivated *Leptospira*, suggesting that next to TLR4, also other TLRs may be involved. The observed activation of canine TLR2-expressing reporter cells by inactivated *Leptospira* strains indicates that TLR2 could be one of these TLRs. Next, we analyzed TLR2 and TLR4 activating capabilities by the same *Leptospira* strains using human and mouse TLR-expressing reporter cells. Inactivated *Leptospira* and leptospiral LPS activated not only mouse, but also human TLR4 and this activation was shown to be LPS dependent in both cases. Additionally, inactivated *Leptospira* activated mouse and human TLR2-expressing reporter cell lines. In our study, we could not identify significant species differences in the recognition of *Leptospira* by TLR2 and TLR4 between dog, human and mouse. Lastly, we show that these inactivated *Leptospira* strains are recognized by both mouse and human TLR5 reporter cells only after exposure to additional heat-treatment. Unfortunately, we were not able to confirm this in the canine system. Our data show that TLR2 and TLR4 are involved in the recognition of *Leptospira* strains used in the production of canine *Leptospira* vaccines. This study contributes to the understanding of *Leptospira*-induced innate immune responses in dogs, humans, and mice. Future studies are needed to further explore the role of canine TLR2, TLR4 and TLR5 in the induction of vaccine-mediated immunity against *Leptospira*.

## Introduction

Pathogenic *Leptospira* spirochetes are the causative agents of leptospirosis, an emerging global zoonosis affecting 1.03 million people annually worldwide (1). Besides being a serious human infection, leptospirosis also induces severe illness in companion and production animals such as dogs, cattle, sheep, swine and horses (2). *Leptospira* cause a systemic infection with a variety of disease manifestations, ranging from asymptomatic infection and mild subclinical symptoms to multiple organ failure or death (3). In dogs, symptoms may include fever, anorexia, vomiting, acute kidney and renal injury, pulmonary hemorrhage, uveitis, myositis, and reproductive failure and death (2, 4). *Leptospira* colonize renal tubules of asymptomatic reservoir hosts such as rats and mice and are shed through urine in the environment where they can survive for months. Susceptible animals, such as dogs, become infected through direct contact with urine from infected animals or indirectly through contaminated water or soil. *Leptospira* then enter the bloodstream *via* damaged skin or mucosal membranes and disseminate to distant organs causing a febrile illness and organ dysfunction (3).

More than 300 serovars of *Leptospira* have been identified based on the carbohydrate structure of the lipopolysaccharide (LPS) (5). In addition, recent whole-genome sequencing studies revealed a large species heterogeneity and proposed several new, potentially pathogenic, *Leptospira* species (6–8). This large species diversity makes precise diagnostics and the development of a universal vaccine challenging. Canine *Leptospira* vaccines have been licensed since 1960 and consist of chemically or physically inactivated whole bacterial cell vaccines (bacterins) (9). Protection induced by most of these bacterin vaccines is relatively short-lasting and considered to be serovar-specific (9–11). Nevertheless, vaccine-induced cross-protective immunity against a serovar not included in the vaccine was recently described (12). Most canine *Leptospira* vaccines currently used contain four whole-inactivated *Leptospira* strains representing serogroups Canicola, Icterohaemorrhagiae, Grippotyphosa and Australis (11). Because of the health risks for the dog and the zoonotic potential, annual vaccination of domestic dogs is recommended (2).

Toll-like receptors (TLRs) are a family of type I transmembrane proteins which recognize microbe-associated molecular patterns (MAMPs) such as LPS, peptidoglycan, lipoproteins, flagellin, single-stranded RNA, viral and bacterial DNA (13). Most cells, including epithelial cells, fibroblasts and immune cells express TLRs on their surface or intracellularly. Ligand binding by TLRs activates a complex network of signal transduction proteins, which induce the secretion of pro-inflammatory cytokines and antimicrobial peptides, resulting in the recruitment and activation of neutrophils, macrophages and dendritic cells, and ultimately the activation of the adaptive immune response (13). TLRs are evolutionary conserved and ten and twelve functional TLRs were identified in humans and mice, respectively (14). Similarly, in dogs the expression of TLR1 (15), TLR2 (16, 17), TLR3 (18, 19), TLR4 (20, 21), TLR5 (21–23), TLR6 (15, 24), TLR7 (19), TLR8 (25) and TLR9 (26) has been described. However, most canine TLR studies have focused only on gene sequence analysis and tissue mRNA or protein expression. In addition, functional activation of canine TLR5 in HEK293 cells by a recombinant FliC (22) and flagellin, purified from *Salmonella enterica* serovar Typhimurium (23) has been reported.

*Leptospira* are Gram-negative, endoflagellar bacteria characterized by an inner membrane and a peptidoglycan layer covered by an outer membrane consisting of phospholipids and surface-exposed components such as LPS and lipoproteins (5). Previous studies suggest that whole, heat-killed *Leptospira* or their purified LPS signal through human and mouse TLR2 (27, 28), even though TLR4 is considered the canonical receptor mediating LPS-induced responses in most species, while TLR2 is considered the canonical receptor mediating lipoprotein induced responses (14). In addition, Nahori *et al.* also showed that while mouse TLR4 recognizes leptospiral LPS, human TLR4 activation was very limited (28). Furthermore, the most abundant leptospiral lipoprotein, LipL32 (29), was shown to activate human (27) and mouse TLR2 (30) through TLR2/TLR1, specific for triacylated lipoproteins (28). Together, these studies suggest that TLR2 in mice and humans are involved in the recognition of different *Leptospira* strains and that TLR4 activation by *Leptospira* may differ across species. In contrast, the role of TLR5, the flagellin receptor (14), in the recognition of *Leptospira* is less clear. Only recently, an asymmetric protein sheath, surrounding the core leptospiral flagellar filament, was discovered (31), suggesting that live, intact *Leptospira* may evade recognition by TLR5 (32). The activation of TLR2, TLR4 and TLR5 by inactivated *Leptospira* strains included in canine *Leptospira* vaccines has not been investigated in any species, including the dog.

In the present study, we investigated whether inactivated *L. interrogans* (serogroups Canicola, Icterohaemorrhagiae and Australis) or *L. kirschneri* (serogroup Grippotyphosa) strains, included in several European canine *Leptospira* vaccines (11), are recognized by canine TLR2 and TLR4. Using the endotoxin neutralizing agent polymyxin B, and TLR4 antagonist *Rhodobacter sphaeroides* LPS (RS-LPS), we show that IL-1β production and *Leptospira* uptake in canine monocyte-derived dendritic cells (moDC) are dependent on leptospiral LPS. Furthermore, using reporter cells expressing canine TLR2 we show that these *Leptospira* strains activate dog TLR2. In addition, to investigate the potential species specificity of TLR responses induced by these four *Leptospira* strains, activation of human and mouse TLR2, TLR4 and TLR5 was studied using reporter cell lines expressing these TLRs. Our results reveal that both mouse and human TLR4 respond to inactivated *Leptospira* as well as to purified leptospiral LPS. Similar to canine TLR2, both mouse and human TLR2 were activated by these *Leptospira* strains. Lastly, we show that both mouse and human TLR5 responded similarly to heat-treated *Leptospira*. In this study, we investigated whether canine TLR2, TLR4 and TLR5 are involved in the recognition of *Leptospira* strains used in canine *Leptospira* vaccine formulations and compared the activation with responses of their human and mouse TLR counterparts.

## Materials and Methods

### Inactivated *Leptospira* Strains

Chemically inactivated pathogenic *L. interrogans* serogroup Canicola serovar Portland-vere (strain Ca-12-000), *L. interrogans* serogroup Icterohaemorrhagiae serovar Copenhageni (strain Ic-02-001), *L. interrogans* serogroup Australis serovar Bratislava (strain As-05-073) and *L. kirschneri* serogroup Grippotyphosa serovar Dadas (strain Gr-01-005) were kindly provided by a pharmaceutical company that is part of the VAC2VAC consortium (http://www.vac2vac.eu/), hereafter referred to as company B. To estimate bacterial cell count, the optical density of these bacterial preparations was measured at 600 nm on an Ultrospec 200 spectrophotometer (Amersham Pharmacia Biotech, Inc.) using Ellinghausen-McCullough-Johnson-Harris (EMJH)-based medium (leptospiral culture medium) as a reference (provided by company B). To allow comparison between all four bacterial preparations, *Leptospira* suspensions were diluted to the same OD_600_ (0.519) in leptospiral culture medium. Subsequently, cells were stimulated with the resulting suspensions at 1:25 dilution throughout this study, unless specified otherwise.

### Leptospiral LPS Purification and Quantification

Leptospiral LPS was extracted from the whole-inactivated bacterial preparations mentioned above by the hot phenol/water method, as described previously (33, 34). Then, the aqueous phase of phenol/water extractions was selected for purification of leptospiral LPS by solid phase extraction on C8 reversed-phase cartridges (34). LPS concentration was determined by analyzing the content of 3-hydroxydodecanoic acid in the samples by gas chromatography, as described earlier (35). All leptospiral LPS preparations were diluted to the lowest concentration (0.056 µmol/ml) in culture medium and 1:1000 dilution of the resulting suspensions was used to stimulate cells throughout this study unless specified otherwise. SDS-PAGE was performed to assess lipoprotein contamination of isolated leptospiral LPS. Samples were separated in a NuPAGE™ 4-12% Bis-Tris SDS-PAGE gel (Thermo Scientific) and stained with Coomassie dye R-250 using the ready-to-use colorimetric Imperial™ Protein Stain (Thermo Scientific). The Coomassie-stained gel image was digitally recorded using an Epson scanner.

### MS Analysis

The lipid A of purified leptospiral LPS were analyzed by negative-ion electrospray ionization (ESI) Fourier transform (FT) MS with in-source collision-induced dissociation (CID) on an LTQ Orbitrap XL instrument (Thermo Scientific). In-source CID of LPS was performed at a high potential difference of 100 V which produced intense fragment ions corresponding to intact lipid A moieties, originating from the rupture of the labile linkage between the nonreducing lipid A glucosamine and Kdo (36). The LPS was infused into the mass spectrometer by using an automated nanoflow HPLC capillary column switching system (nLC) described previously for protein and peptide analysis (37). The nLC system was comprised of trapping (2 cm x 100 μm i.d.) and analytical (20 cm x 50 μm i.d.) columns containing reversed-phase POROS R2 particles (Thermo Scientific) packed in-house. The solvents water/triethylamine/acetic acid (100/0.03/0.01, vol%) pH=8.6 (solvent A) and 2-propanol/triethylamine/acetic acid (70:30:0.03:0.01, vol%) pH=8.6 (solvent B) were mixed and delivered for nLC-ESI-FT-MS as follows. Trapping stage: solvent B was kept at 30 vol% for 15 min; analytical separation: solvent B was increased directly to 45 vol%, then linearly from 45 to 75 vol% over 15 min and then kept at 100 vol% B for 10 min; column re-equilibration: solvent B was directly decreased to 30 vol% and then kept at 30 vol% for 6 min (38). Lipid A mass spectra generated by in-source CID nLC-ESI-FT MS of LPS were averaged over the whole elution time window of LPS molecules. The suggested Lipid A compositions are based on the chemical structure of leptospiral lipid A reported previously (39). The given mass-to-charge ratios refer to monoisotopic molecular masses.

### Canine Monocyte-Derived Dendritic Cell Culture and Stimulation

Peripheral blood mononuclear cells (PBMC) were isolated at room temperature (RT) by density gradient centrifugation from buffy coats obtained from the Companion animal clinics, Faculty of Veterinary Medicine, Utrecht University under owner’s consent. Briefly, buffy coats were diluted 2x with Dulbecco’s Phosphate-buffered saline without calcium and magnesium (DPBS-/-; Corning), layered onto Histopaque-1077 (Sigma Life Science) and centrifuged 30 min at 800xg with slow acceleration and brake. The cloudy interphase containing PBMC was transferred to a new tube and washed twice in culture medium consisting of RPMI-1640 GlutaMAX (Gibco) supplemented with 5% heat inactivated fetal bovine serum (FBS; Bodinco BV), 50 U/ml Penicillin and 50 µg/ml Streptomycin (Gibco). PBMC were counted on the NucleoCounter (ChemoMetec) and either cryopreserved or directly used for CD14^+^ monocyte isolation by magnetic activated cell sorting (MACS). Briefly, PBMC were labeled with the monoclonal mouse anti-human CD14 TUK4 antibody (Bio-Rad) for 30 min on ice. Cells were washed with MACS buffer consisting of DPBS-/- supplemented with 0.5% heat inactivated FBS and 2 mM UltraPure EDTA (Invitrogen). PBMC were labeled with anti-mouse IgG Microbeads (Miltenyi Biotec) and CD14^+^ monocytes were isolated following manufacturer’s protocol. For differentiation into moDC, 0.75 x 10^6^/ml CD14^+^ monocytes were cultured in 12- or 24-well culture plates (Corning) in culture medium supplemented with 0.05 mM β-mercaptoethanol (Sigma Life Science), 20 ng/ml recombinant canine interleukin 4 (rcIL-4) and 20 ng/ml recombinant canine granulocyte-macrophage colony stimulating factor (rcGM-CSF) (both R&D Systems). Culture medium containing 0.05 mM β-mercaptoethanol, 40 ng/ml rcIL-4 and 40 ng/ml rcGM-CSF was added to cells 72 hours after seeding. Cells were incubated for another 72 hours before stimulation with the following stimuli: 1 μg/ml *E. coli* K12 LPS Ultrapure, 1 μg/ml Pam3CSK4 or 1 μg/ml *Salmonella typhimurium* flagellin (FLA-ST; all *In vivo*Gen) as positive controls for TLR4, TLR2 and TLR5 respectively, whole-inactivated bacteria or purified LPS of *L. interrogans* (serogroup Canicola, Icterohaemorrhagiae or Australis) or *L. kirschneri* serogroup Grippotyphosa. To determine the role of leptospiral LPS in the induction of cytokine release, *Leptospira* or purified leptospiral LPS were pre-incubated with LPS neutralizing reagent polymyxin B (40) (Sigma Life Science) for 1-3 hours at 37°C and 5% CO_2_ resulting in a final concentration of 50 µg/ml polymyxin B after addition to the cells. In addition, to determine the role of TLR4 signaling, moDC were treated with 50 µg/ml *Rhodobacter sphaeroides* LPS (RS-LPS; *In vivo*Gen), an antagonist of human and mouse TLR4 (41), for 1 hour at 37°C and 5% CO_2_ before stimulation with *Leptospira* or LPS.

### RT-qPCR

To determine mRNA expression of IL-1β, IL-6, and IL-12p40 cytokines in canine moDC, cells were lysed in RLT buffer (Qiagen) 6, 18 or 24 h after stimulation and stored at −20°C until further processing. After thawing, total RNA was isolated with the RNeasy Kit (Qiagen). Next, cDNA was synthesized using 200 ng of total RNA and the iScript cDNA Synthesis kit (Bio-Rad) according to the manufacturer’s instructions. RT-qPCR reactions were performed with 300 nM primers listed in **Table 1** and iQ SYBR Green Supermix on a CFX Connect qPCR detection system (both Bio-Rad). RT-qPCR reactions were performed at 95°C for 3 min, followed by 39 cycles of 95°C for 15 s and 55°C for 30 s, and a melting curve from 55°C to 95°C with 0.5°C increments for 5 seconds. Amplification efficiency (90–110%) was evaluated using serial dilutions of a standard cDNA before testing the samples (data not shown). RT-qPCR reactions were performed in triplicate for each sample and analyzed with CFX Maestro software (Bio-Rad). Fold change in gene expression over time upon stimulation was assessed using unstimulated cells (t = 6 h) as a reference time point and normalized to gene expression levels of the housekeeping gene glyceraldehyde 3-phosphate dehydrogenase (GAPDH). Each condition was performed in duplicate or triplicate and data shown represent mean values from two or three independent experiments.

**Table 1:** RT-qPCR primers.

Primer name	Primer sequence (5’to 3’)	Reference
**IL-1β F**	TCTCCCACCAGCTCTGTAACAA	(42)
**IL-1β R**	GCAGGGCTTCTTCAGCTTCTC	(42)
**IL-6 F**	TCCTGGTGATGGCTACTGCTT	(42)
**IL-6 R**	GACTATTTGAAGTGGCATCATCCTT	(42)
**IL-12p40 F**	CAGCAGAGAGGGTCAGAGTGG	(42)
**IL-12p40 R**	ACGACCTCGATGGGTAGGC	(42)
**GAPDH F**	GATGGGCGTGAACCATGAGA	(43)
**GAPDH R**	TGGTCATGGATGACTTTGGCT	(43)

### IL-1β Protein Detection

Canine moDC were incubated for 48 h with leptospiral culture medium as negative control (1:25), whole inactivated serogroup Canicola (1:25), and its purified LPS (1:1000) as described in section “Canine monocyte-derived dendritic cell culture and stimulation”. Supernatants were stored at -20°C until analysis. An ELISA kit for canine IL-1β (Kingfisher Biotech, Inc) was used in in-house developed cytokine bead assay using the Magpix system (Luminex XMAP) according to the manufacturer’s instruction. The cytokine concentration in the supernatants of stimulated cells was calculated using the standard provided in the kit. The MFI data were analyzed using a 5-parameter logistic method (xPONENT software, Luminex, USA).

### Flow Cytometry

Canine moDC were harvested by several washes with DPBS-/- or 10 min incubation in enzyme-free PBS-based Cell Dissociation buffer (Gibco) and stained with Zombie NIR viability dye (Biolegend) in DPBS-/- for 15 min at RT in the dark. Cells were then labelled with mouse anti-dog CD1a8/CD1a9 CA13.9H11 and mouse anti-dog CD86 CA24.3E4 monoclonal antibodies (mAbs) (both University of California, Davis, USA) in 50 µl FACS buffer (DPBS-/-, 2 mM UltraPure EDTA (Invitrogen) 2% heat inactivated FBS) supplemented with 2% normal dog plasma (Utrecht University) for 30 min on ice, followed by rat anti-mouse IgG1 PerCP-Cy5.5 and rat anti-mouse IgG2a PE (both Biolegend) mAb staining. After blocking with 5% mouse serum, moDC were labelled with mouse anti-dog CD11c biotin CA11.7D1 (University of California, Davis, USA), cross-reactive mouse anti-human CD14 Pacific Blue TUK4, cross-reactive mouse anti-human CD40 Alexa Fluor 647 LOB7/6 (both Bio-Rad), cross-reactive hamster anti-mouse CD80 PE-Cy7 16-10A1 (eBioscience) and cross-reactive mouse anti-rat MHC-II FITC (Utrecht University, the Netherlands) mAbs. Lastly, moDC were stained with Streptavidin V500 (BD Bioscience) for 15 min on ice. Cells were washed twice with 150 µl FACS buffer and centrifuged 3 min at 400xg between the stainings. The expression of surface markers was analyzed on a CytoFLEX LX flow cytometer (Beckman Coulter) at the Flow Cytometry and Cell Sorting Facility, Faculty of Veterinary Medicine, Utrecht University.

### Phagocytosis Assay

Canine moDC were cultured at 0.75 x 10^6^/ml on ethanol-cleaned 12-mm glass coverslips (Waldemar Knittel Glasbearbeitungs GmbH, Germany) in a 24-well culture plate as described in section “Canine monocyte-derived dendritic cell culture and stimulation”. Suspensions of the inactivated *Leptospira* were centrifuged 15 min at 3095xg and washed twice with 0,1 M bicarbonate buffer pH 8,3. After centrifugation, the pellet was suspended in 1 ml 0,1 M bicarbonate buffer and labelled with 50 µg/ml Atto 488 NHS ester (Sigma-Aldrich, Germany) for 1 hour at RT under constant shaking. *Leptospira* were then centrifuged as above and non-reacted NHS groups were quenched with 20 mM NH4Cl in DPBS-/- for 10 min at RT under constant shaking. *Leptospira* were washed three times in DPBS-/- and the bacterial concentration was estimated on a CytoFLEX LX flow cytometer. Fluorescently-labeled *Leptospira* were then incubated with 10% normal dog plasma (Utrecht University) for 20 min at 37°C in a water bath, centrifuged as above and resuspended in culture medium. To determine the role of leptospiral LPS and TLR4 in bacterial uptake, moDC were stimulated with fluorescently-labeled *Leptospira* in 1:3 ratio in presence of polymyxin B or RS-LPS as described in section “Canine monocyte-derived dendritic cell culture and stimulation”. To demonstrate the specificity of bacterial uptake, moDC were incubated at 4°C as well. To stop the phagocytosis process, cells were briefly incubated on ice and fixed with 4% paraformaldehyde (PFA; Electron Microscopy Sciences, USA) by 30 min incubation at RT. After fixation, cells were washed twice in DPBS-/- and glass cover slips were stained for confocal microscopy as described in the next section. The remaining cells in the culture plate were incubated with 20 mM NH4Cl in DPBS-/- for 20 min at RT, followed by 5 min incubation with 0.1% Trypan blue at 4°C before staining for flow cytometry according to the method described above. Cells were labeled with mouse anti-dog MHC II mAb CA2.1C12 (University of California, Davis, USA) followed by anti-mouse IgG1 BV421 mAb (Biolegend) and analyzed on a CytoFLEX LX flow cytometer at the Flow Cytometry and Cell Sorting Facility, Faculty of Veterinary Medicine, Utrecht University.

### Immunofluorescence Microscopy

Twelve-mm glass coverslips with moDC were transferred inverted onto parafilm and stained with mouse anti-canine MHC-II CA2.1C12 (University of California, Davis, USA) and mouse anti-human TLR4 HTA125 (Invitrogen) mAbs for 30-45 min at RT in blocking buffer consisting of DPBS-/-, 0.1% saponin (Sigma) and 2% bovine serum albumin (Sigma). Next, moDC were stained with anti-mouse IgG1 Alexa Fluor 647 and anti-mouse IgG2a Alexa Fluor 568 mAbs (both Invitrogen) as above. In addition, the nuclei were stained with 240 nM 4′,6-diamidino-2-phenylindole (DAPI) (Thermo Fisher Scientific) for 1 min in DPBS-/-. Samples were washed three times in blocking buffer between the staining steps. After the last wash, coverslips were rinsed with distilled water and mounted in Prolong Diamond (Thermo Scientific) on frosted end microscope slides (Menzel Glaser GmbH & Co KG, Germany). To visualize internalization of *Leptospira* in canine moDC, images of Prolong Diamond embedded slides were acquired on a NIKON A1R confocal system equipped with 405, 488, 561 and 647 nm lasers, using a 100x Plan Apo oil objective (NA1.45). A sequential imaging procedure was used to collect stack series with 150 nm step size using a quad band dichroic and 475/30, 515/30, 595/50 and 700/75 nm emission filters with pixel size of 120 nm and 58.75 µm pinhole. Images were processed in NIS elements 5.3 software (NIKON). Acquired stack series were denoised with the denoise.ai module, deconvolved (according to the blind algorithm with axial psf correction) and maximum intensity projections were performed. Contrast was optimized to highlight both the spiral-shaped appearance of *Leptospira* as well as the spherical shape appearance (gamma correction 0.7 for all channels, with standardized intensity settings).

### Generation of cTLR2 Expression Plasmids and cTLR2 Expressing Cells

To investigate the activation of canine TLR2 by *Leptospira*, TLR2 was cloned from canine PBMC. Peripheral blood samples were obtained from a one-year old healthy female mixed breed dog with owner’s consent. Blood was collected by venipuncture into LH BD Vacutainer tubes (Becton-Dickinson) and PBMC were isolated by density gradient centrifugation as described in section “Canine monocyte-derived dendritic cell culture and stimulation”. Total RNA was isolated from PBMC using the RNeasy Mini Kit and 500 ng total RNA was used for cDNA synthesis using the SuperScript III First-Strand Synthesis System (Invitrogen) following the manufacturer’s protocol. Canine TLR2 (cTLR2) was amplified by PCR using the AccuPrime Taq DNA polymerase high-fidelity kit (Invitrogen), 1 μl of cDNA as a template and Canine TLR2 F and Canine TLR2 R primers containing restriction enzyme sites for further subcloning (**Table 2**). The PCR reaction was performed at 94°C for 2 min, followed by 34 cycles of 94°C for 30 s, then 68°C for 30 s and 68°C for 2 min 50 s, and final incubation at 72°C for 5 min. The resulting PCR products were ligated into the pGEM-T-Easy shuttle vector (Promega). Subsequently, pGEM-T-Easy-cTLR2 plasmid was digested with BamHI and Sall restriction enzymes, and the BamHI/SalI cDNA fragment encoding full length cTLR2 (aa 1-786) was inserted into BamHI/SalI digested expression plasmid pDUO2 (*In vivo*Gen) following the Rapid DNA ligation kit protocol (Thermo Fisher Scientific). Presence and integrity of the cTLR2 insert in the pDUO2-cTLR2 miniprep was confirmed by PCR and Sanger sequencing using all four primers listed in **Table 2**. For transfection, pDUO2-cTLR2 plasmid DNA was subsequently isolated using the NucleoBond Xtra midiprep kit (Macherey Nagel).

**Table 2:** Primers used for cloning of canine TLR2.

Primer name	Primer sequence (5’to 3’)	Reference
**Canine TLR2 F**	GGATCCTCGTCACCTCCCGGGTT	GenBank AB189639.1
**Canine TLR2 R**	GTCGACCTGCAAAGGACAGTGCGTTC	GenBank AB189639.1
**Canine TLR2 int F**	GTACGGAGGTTGCATATTCCACAC	This study
**Canine TLR2 int**	CCTTTTGGCCTGGAGCCAGG	This study

Introduced restriction sites are underlined.

To generate reporter cells expressing cTLR2, 2 x 10^6^ HEK-Blue-Null1 cells (*In vivo*Gen) were seeded in a 25 cm2 flask (Corning) in HEK-Blue culture medium consisting of DMEM (Gibco) supplemented with 10% heat-inactivated FBS, 50 U/ml Penicillin and 50 µg/ml Streptomycin and incubated 2 hours at 37°C and 5% CO_2_. Twelve μg of pDUO2-cTLR2 plasmid DNA was incubated with Lipofectamine 2000 (Invitrogen) in DMEM for 20 min at RT before cell transfection. After 48 hours, the medium was replaced with fresh HEK-Blue culture medium and cells were incubated additionally for 24 hours before harvesting. To select for cTLR2-expressing reporter cells (referred to as HB-cTLR2 throughout this manuscript), transfected cells were maintained in HEK-Blue culture medium supplemented with 100 μg/ml Zeocin and 100 μg/ml Hygromycin B (both *In vivo*Gen), but not cloned by limiting dilution or cell sorting.

### TLR Sequence Homology and Structural Analysis

Human (Uniprot ID O60603) and mouse (Uniprot ID Q9QUN7) TLR2 amino acid sequences were obtained from Uniprot (https://www.uniprot.org/). Pairwise sequence alignments with canine TLR2 were performed with EMBOSS Needle (https://www.ebi.ac.uk/Tools/psa/emboss_needle/) using the default settings. The visualization depicting structural similarities of dog and human TLR2 extracellular domain was done with PyMOL Molecular Graphics System Version 1.3 (https://pymol.org/2/). The crystal structure of human TLR2 extracellular domain (PDB identifier 6NIG) was used generate the homology model.

### TLR Reporter Assay

To investigate the activation of TLRs by *Leptospira*, HEK-Blue cells expressing canine TLR2 (generated in this study), mouse and human TLR2, TLR4 or TLR5 (all *In vivo*Gen), and the parental HEK-Blue-Null1 cell line (*In vivo*Gen) were maintained in HEK-Blue culture medium at 37°C in a humidified atmosphere of 5% CO_2_. Cells were cultured in presence of selective antibiotics as recommended by the manufacturer and passaged two times per week at 80-90% confluency.

HEK-Blue cells expressing mouse or human TLR2, TLR4 or TLR5; or canine TLR2 were seeded at 5 x 10^4^ cells per well in flat bottom 96-well culture plates (Corning) in a total volume of 100 µl HEK-Blue culture medium and incubated over-night. Cells were stimulated with 100 µl *E. coli* K12 LPS Ultrapure, Pam3CSK4 or *Salmonella typhimurium* flagellin (FLA-ST) as positive controls for TLR4, TLR2 and TLR5 respectively, leptospiral culture medium as negative control, or whole-inactivated bacteria or purified LPS of *L. interrogans* (serogroup Canicola, Icterohaemorrhagiae or Australis) or *L. kirschneri* serogroup Grippotyphosa. The parental HEK-Blue-Null1 cell line was used as the control to check for the endogenous TLR activation (**Supplementary Figure S5**). To block the innate immune stimulating properties of leptospiral LPS, whole-inactivated *Leptospira* or purified leptospiral LPS were incubated with polymyxin B for 1 hour at 37°C before stimulating the cells. To remove potential lipoprotein contaminants, purified leptospiral LPS was treated with 1 mg/ml Proteinase K (Sigma Life Science) at 37°C over-night. Subsequently, proteinase K was inactivated by 10 min incubation at 95°C before cell stimulation. Furthermore, whole-inactivated *Leptospira* were incubated for 30 min at 85°C and 300 rpm as described (32) before stimulating mouse or human TLR5 expressing HEK-Blue cells. In all experiments, cells were stimulated for 20-24 hours at 37°C and 5% CO_2_. Activation of HEK-Blue reporter cells was determined by mixing 20 μl of each cell supernatant with 180 μl QUANTI-Blue Detection medium (*In vivo*Gen), followed by incubation at 37°C for up to 3 hours. Absorption at 630 or 650 nm was then measured spectrophotometrically using a Model 550 Microplate Reader (Bio-Rad).

### Statistical Analysis

Statistical analyses were performed using GraphPad Prism 9 software (GraphPad Software Inc., San Diego, CA, USA). The normality of the data was assessed in GraphPad Prism with Q-Q plots or the Shapiro-Wilk normality test. Relative gene expression data were log-transformed to create normally distributed data. A p-value of <0.05 was considered statistically significant.

## Results

### Inactivated *Leptospira* Upregulate Expression of Pro-Inflammatory Cytokines in Canine moDC

**Figure 1:**
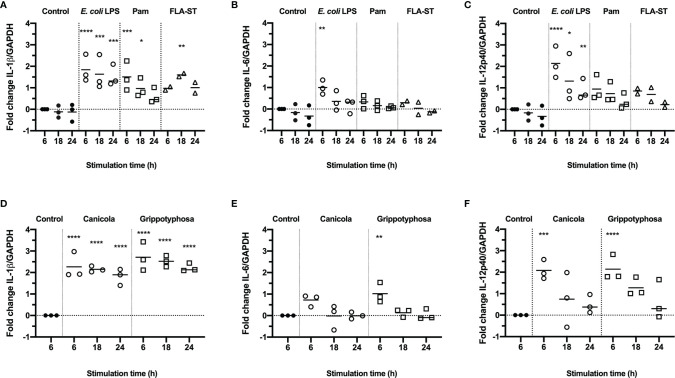
Production of inflammatory cytokines in canine moDC stimulated with TLR agonists and *Leptospira*. RT-qPCR was performed on mRNA from canine moDC stimulated for 6, 18 and 24 h with TLR agonists *E. coli* LPS, Pam3CSK4 (Pam) or flagellin (FLA-ST) **(A–C)** or whole-inactivated *L. interrogans* serogroup Canicola or *L. kirschneri* serogroup Grippotyphosa **(D–F)**. IL-1β **(A, D)**, IL-6 **(B, E)**, and IL-12p40 **(C, F)** gene expression was normalized to GAPDH and fold change in expression relative to the unstimulated control (Control; t=6) is shown. Data represent mean values from three independent experiments. Each experiment was performed using two replicates per condition. A mixed-effect analysis combined with Dunnett’s multiple comparisons test was used to test for statistical significance between the stimulated sample and unstimulated control at 6 h; *p < 0.05, **p < 0.01, ***p < 0.001, ****p < 0.0001.

To assess whether canine monocyte derived dendritic cells (moDC) are suitable to study innate immune responses induced by *Leptospira in vitro*, canine CD14^+^ monocytes were differentiated into CD11c^+^ MHC class II^+^ CD80^+^ CD86^+^ cells in the presence of rcIL-4 and rcGM-CSF (**Supplementary Figure S1**). Subsequently, canine moDC were stimulated with *E. coli* LPS, Pam3CSK4 (Pam) or *Salmonella typhimurium* flagellin (FLA-ST) as positive controls (**Figures 1A-C**), or whole-inactivated *L. interrogans* serogroup Canicola or *L. kirschneri* serogroup Grippotyphosa (**Figures 1D-F**) for 6, 18 and 24 hours. The mRNA expression of pro-inflammatory cytokines IL-1β (**Figures 1A, D** **)**, IL-6 (**Figures 1B, E** **)** and IL-12p40 (**Figures 1C, F** **)** was assessed by RT-qPCR. Stimulation with Canicola and Grippotyphosa as well as all TLR ligands significantly upregulated IL-1β expression at different time points, when compared to the unstimulated control at 6 h. IL-6 and IL-12p40 mRNA expression was significantly upregulated only after stimulation with *Leptospira* serogroups and *E. coli* LPS (**Figure 1**). These results show that TLR2, TLR4 and TLR5 ligands as well as inactivated *Leptospira* induce innate immune responses in canine moDC rapidly.

### IL-1β Expression and the Uptake of *Leptospira* by Canine moDC Is Partially Dependent on Leptospiral LPS and TLR4 Activation

**Figure 2:**
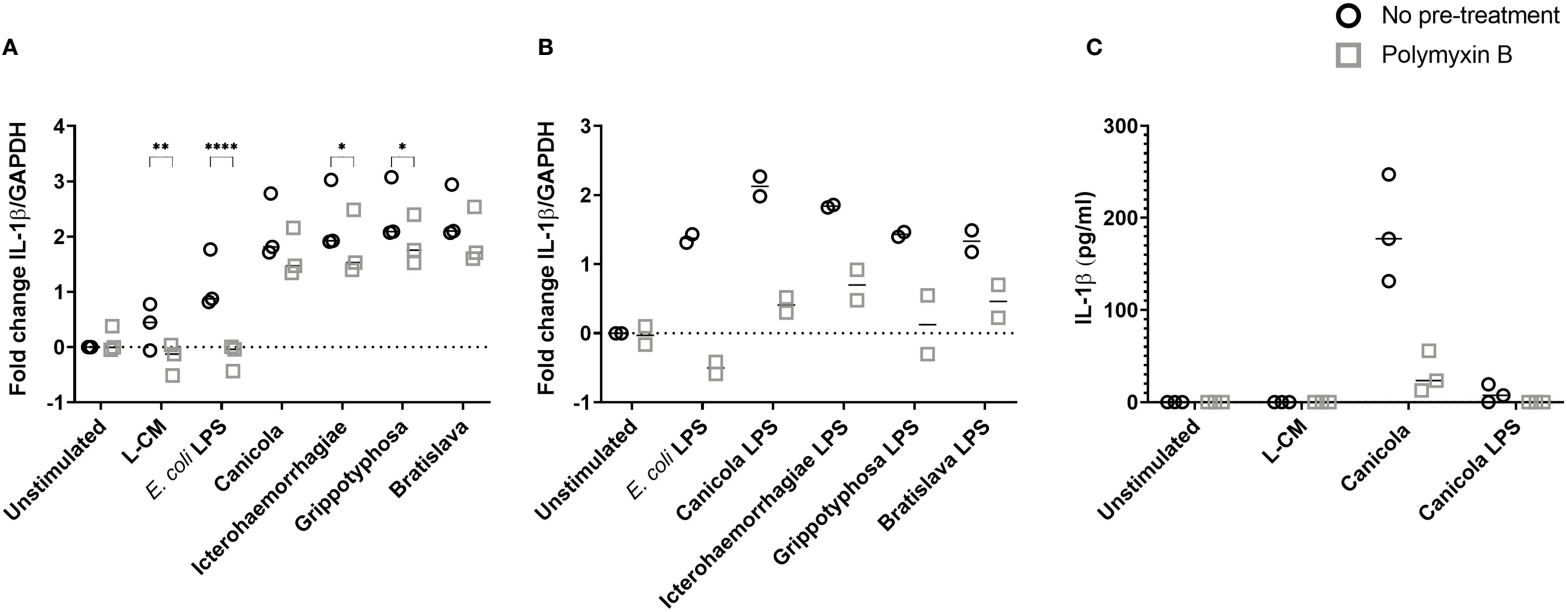
IL-1β production following stimulation with inactivated *Leptospira* is partially dependent on leptospiral LPS in canine moDC. **(A, B)** RT-qPCR was performed on mRNA from canine moDC stimulated for 6 (h). **(A)** Cells were incubated with *E. coli* LPS, leptospiral culture medium (L-CM) as positive and negative controls, respectively, whole-inactivated *L. interrogans* serogroup Canicola, Icterohaemorrhagiae or Bratislava or *L. kirschneri* serogroup Grippotyphosa (circle). In addition, moDC were incubated with *Leptospira* pre-treated with polymyxin B (square). IL-1β gene expression was normalized to GAPDH and fold change in expression relative to the unstimulated control is shown. Data represent mean values from three independent experiments. Each experiment was performed using two replicates per condition. A mixed-effect analysis combined with Šidák’s multiple comparisons test was used to test for statistical significance between the untreated and polymyxin B treated sample; *p < 0.05, **p < 0.01, ****p < 0.0001. **(B)** Cells were incubated with *E. coli* LPS as control or LPS isolated from four *Leptospira* strains shown in **A** (circle). In addition, moDC were stimulated with leptospiral LPS pre-treated with polymyxin B (square). IL-1β gene expression was normalized to GAPDH and fold change in expression relative to the unstimulated control is shown. Data represent mean values from two independent experiments. Each experiment was performed using two replicates per condition. **(C)** IL-1β protein was measured in the supernatants of canine moDC incubated for 48 h with leptospiral culture medium as control, whole inactivated serogroup Canicola or its purified LPS (circle), or polymyxin B treated stimuli (square). The experiment was performed once in three technical replicates per stimulation.

Because the LPS is considered the main leptospiral MAMP, we sought to determine the role of leptospiral LPS in the induction of the inflammatory responses observed in canine moDC (**Figure 2**). Canine moDC were stimulated with whole-inactivated *L. interrogans* serogroup Canicola, Icterohaemorrhagiae, and Bratislava or *L. kirschneri* serogroup Grippotyphosa in presence of endotoxin neutralizing reagent polymyxin B, while *E. coli* LPS was used as a positive control (**Figure 2A**). IL-1β mRNA expression increased after 6 h stimulation with all four whole-inactivated *Leptospira* strains and *E. coli* LPS when compared to the unstimulated moDC. Polymyxin B treatment completely abolished IL-1β expression in *E. coli* LPS stimulated moDC. In contrast, although polymyxin B treatment significantly reduced the level of IL-1β mRNA expression in Icterohaemorrhagiae and Grippotyphosa stimulated cells, the IL-1β mRNA remained upregulated compared to the unstimulated control, indicating that in addition to LPS, also other MAMPs are likely involved in triggering cytokine production in canine moDC.

To further investigate whether the upregulation of IL-1β observed in canine moDC after stimulation with inactivated *Leptospira* is induced by leptospiral LPS, we purified the LPS from whole-inactivated bacteria of *L. interrogans* serogroup Canicola, Icterohaemorrhagiae, and Bratislava and *L. kirschneri* serogroup Grippotyphosa. MS with in-source CID of the purified LPS showed singly deprotonated fragment ion peaks in the range of *m/z* from 1680 to 1760 (**Supplementary Figure S2A**), which compare well to the *m/z* values of previously described leptospiral lipid A structures (39, 44). In particular, a major ion peak of *m/z* 1720.22 or 1720.23 observed in the in-source CID mass spectra of the LPS from *L. interrogans* serogroup Canicola, Icterohaemorrhagiae and Bratislava (**Supplementary Figure S2A**) is consistent with a four amide-linked hexa-acylated lipid A structure containing a methylated phosphate group and four carbon-carbon double bonds in the fatty acids, as previously reported (see structure on top of **Supplementary Figure S2A**) (39, 44). In addition, the latter mass spectra showed two fragment ion peaks of *m/z* 1748.25 or 1748.26 and *m/z* 1692.19 or 1692.20 which are consistent with a leptospiral lipid A structure as described above but with modified fatty acid chains (2 carbons longer or shorter, with calculated *m/z* = 1748.26 and 1692.20, respectively) (**Supplementary Figure S2A**). Overall, isotopic peak distributions were complex due to the presence of a series of subsequent 3 to 4 monoisotopic peaks separated by 2 Da, which is in line with the existence of a variable number (3 to 6) of carbon-carbon double bonds in the structure of the lipid A from *L. interrogans* serogroups Canicola, Icterohaemorrhagiae and Bratislava. Furthermore, minor ion peaks at *m/z* 1708.19 and 1736.22 with 15.99 Da higher molecular masses than those of ion peaks of *m/z* 1692.20 and 1720.23 are consistent with the substitution of fatty acids with an additional hydroxyl group (**Supplementary Figure S2A**). The in-source CID mass spectra of the LPS from *L. kirschneri* serogroup Grippotyphosa displayed ion peaks of several m/z values (e.g., 1744.22, 1718.21, 1716.20, 1690.18), which were equal (within experimental error) to m/z values of ion peaks in the mass spectra of LPS of the three different *L. interrogans* serogroups described above, pointing towards similar lipid A composition and structural heterogeneity of LPS from *L. kirschneri* serogroup Grippotyphosa and LPS from *L. interrogans*. However, ion peaks with the maximum intensity in the in-source CID mass spectra of the LPS from *L. kirschneri* serogroup Grippotyphosa appeared to be shifted by 4 Da to lower m/z values as compared to those in the in-source CID mass spectra of the LPS from the three *L. interrogans* serogroups analyzed in this study. This suggests a higher degree of fatty acid unsaturation of the *L. kirschneri* serogroup Grippotyphosa lipid A compared to lipid A from *L. interrogans* (**Supplementary Figure S2A** **)**.

Next, equimolar amounts of the purified leptospiral LPS preparations described above were used to stimulate canine moDC (**Figure 2B**). After 6 hours, purified LPS preparations from *L. interrogans* serogroup Canicola, Icterohaemorrhagiae, and Bratislava and *L. kirschneri* serogroup Grippotyphosa and *E. coli* LPS upregulated IL-1β mRNA expression compared to the unstimulated moDC. Presence of polymyxin B inhibited IL-1β upregulation induced by LPS derived from all four *Leptospira* serogroups as well as *E. coli* LPS compared to the untreated sample. In addition, polymyxin B inhibited IL-1β protein expression induced by Canicola (**Figure 2C** and **Supplementary Figure S3**). These results confirm that canine moDC specifically respond to leptospiral LPS and provide evidence that other leptospiral MAMPs such as lipoproteins, which are present in *Leptospira*, might induce cytokine production in canine moDC as well.

**Figure 3:**
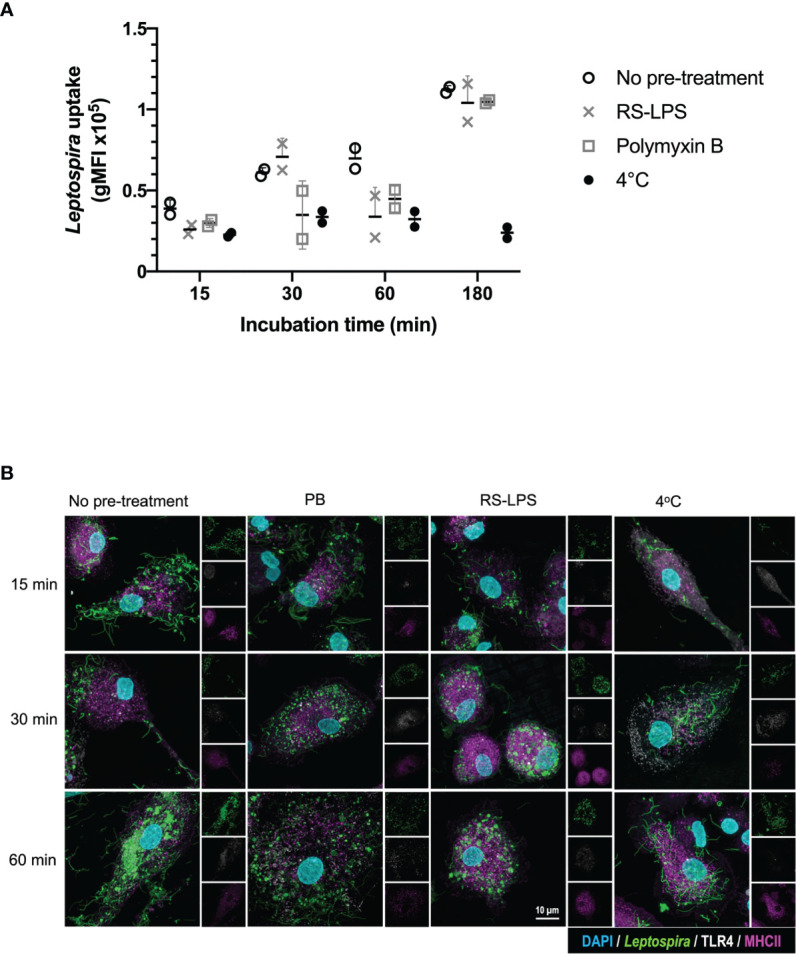
TLR4 signaling is involved in the uptake of inactivated *Leptospira* in canine moDC. MoDC were incubated with Atto 488-labeled inactivated *Leptospira* for 15, 30, 60 or 180 min at 37°C or 4°C. In addition, moDC were pre-treated with RS-LPS or stimulated with *Leptospira* that were pre-incubated with polymyxin B. The experiment was performed using two replicates per condition. **(A)** *Leptospira* uptake was determined by flow cytometry as an increase in Atto 488 geometric mean fluorescence intensity (gMFI) in the MHC II positive gate. The uptake of untreated (open circle), RS-LPS (cross) or polymyxin B (square) pre-treated sample or uptake at 4°C (closed circle) is shown. **(B)** moDC were stained with mouse anti-canine MHC II (magenta) and cross-reactive mouse anti-human TLR4 (white) to visualize bacterial uptake by confocal microscopy. Fluorescent *Leptospira* are shown in green. 4′,6-Diamidino-2-phenylindole (DAPI) (blue) was used to stain the nuclei. 3D images were constructed from stack series with 150 nm step size.

To investigate whether TLR4 is directly involved in the uptake of *Leptospira*, canine moDC were either pre-treated or not with the TLR4 antagonist *Rhodobacter sphaeroides* LPS (RS-LPS) and incubated with fluorescently-labeled inactivated *Leptospira* (**Figure 3** and **Supplementary Figure S4**). *Leptospira* uptake at 37°C enhanced at all incubation times compared to 4°C. The uptake of *Leptospira* in RS-LPS pre-treated moDC was reduced at 15 and 30 min incubation (**Supplementary Figure S4**) and at 60 min incubation compared to the untreated moDC (**Figure 3**). In addition, pre-treatment of *Leptospira* with polymyxin B reduced *Leptospira* uptake at 15, 30 and 60 min incubation. Even though a reduction in bacterial uptake was observed only at 60 min incubation with RS-LPS, the combined results obtained with RS-LPS and polymyxin B pre-incubation suggest that LPS-induced TLR4 signaling is involved in the uptake of *Leptospira* in canine primary cells (**Figures 2**, **3** and **Supplementary Figures S3, S4**).

### Whole-Inactivated *Leptospira* and Purified LPS Activate Mouse and Human TLR4

**Figure 4:**
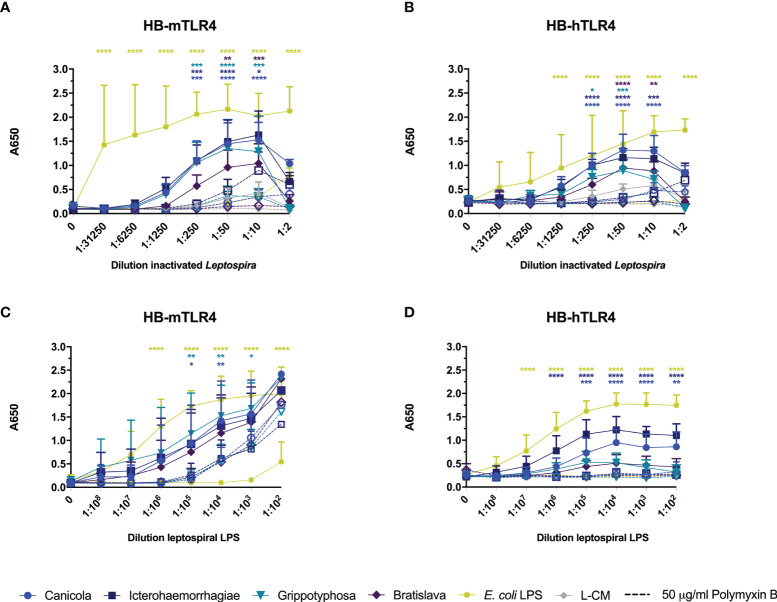
Leptospiral LPS dose-dependently activates mouse and human TLR4. HEK-Blue reporter cell lines expressing **(A, C)** mouse (HB-mTLR4) and **(B, D)** human (HB-hTLR4) TLR4 were stimulated with serially diluted *E. coli* LPS (yellow) or leptospiral culture medium (L-CM; grey) as positive and negative control, respectively or serially diluted whole-inactivated *L. interrogans* serogroup Canicola (circle), Icterohaemorrhagiae (square) or Bratislava (diamond) or *L. kirschneri* serogroup Grippotyphosa (triangle) **(A, B)** or leptospiral LPS isolated from the same serogroups **(C, D)**. Purified leptospiral LPS and *E. coli* LPS were diluted 10-fold with a starting concentration 0.56 nmol/ml and 10 µg/ml, respectively. Dotted lines with open symbols represent stimulations that were pre-treated with polymyxin B, at a final concentration of 50 µg/ml polymyxin B in cell culture. Mean values from three independent assays per cell line are shown. Each assay was performed using two or three replicates per condition. The error bars represent the SD. A mixed-effect analysis combined with Tukey’s multiple comparisons test was used to test for significance between the untreated and polymyxin B treated stimulus at each dilution. In addition, serogroup Canicola and Icterohaemorrhagiae derived LPS appeared as a significantly stronger activator of human TLR4 compared to LPS derived from serogroups Grippotyphosa or Bratislava. *p < 0.05, **p < 0.01, ***p < 0.001, ****p < 0.0001.

Next, we investigated whether inactivated *L. interrogans* serogroup Canicola, Icterohaemorrhagiae, and Bratislava or *L*. *kirschneri* serogroup Grippotyphosa activate reporter cells *via* human and mouse TLR4. HEK-Blue cell lines stably expressing mouse TLR4 (HB-mTLR4; **Figures 4A, C** **)** or human TLR4 (HB-hTLR4; **Figures 4B, D** **)** were stimulated with whole-inactivated *Leptospira* strains (**Figures 4A, B** **)** or serially diluted leptospiral LPS purified from these strains (**Figures 4C, D** **)**. Despite the cytotoxic effect of *Leptospira* at the highest concentration, all four *Leptospira* serogroups activated HB-mTLR4 and HB-hTLR4 cell lines at 1:10 dilution. Further dilution of the inactivated *Leptospira* showed that TLR4 activation was dose-dependent (**Figures 4A, B** **;** solid lines). Although the measured responses were lower in HB-hTLR4 cells compared to HB-mTLR4, serogroup Canicola and Icterohaemorrhagiae appeared as the strongest activators of both TLR4 species. While serogroup Grippotyphosa strongly activated HB-mTLR4, the activation of HB-hTLR4 was lower, indicating possible host preferences by this serogroup. The TLR4 activation by serogroup Bratislava was the lowest in both TLR4 species. In addition, HB-mTLR4 and HB-hTLR4 cells were stimulated with leptospiral culture medium as a negative control. Despite a low TLR4 activation by leptospiral culture medium at 1:10, 1:50 and 1:250 dilution, this was significantly lower compared to TLR4 activation by *Leptospira* (**Figures 4A, B** **)**. Next, to determine whether leptospiral LPS plays a role in the activation of mTLR4 and hTLR4, inactivated *Leptospira* were treated with polymyxin B before HB-mTLR4 and HB-hTLR4 stimulation (**Figures 4A, B** dotted lines). Polymyxin B significantly inhibited mTLR4 activation by all four serogroups at 1:10 and 1:50 dilution. TLR4 activation by the positive control, *E. coli* LPS, was significantly inhibited by polymyxin B as well (**Figure 4A**). Similar to mTLR4, polymyxin B treatment also significantly inhibited the activation of hTLR4 by all four serogroups at 1:50 dilution (**Figure 4B**). This suggests that both mouse and human TLR4 recognize LPS produced by these *Leptospira* strains.

To confirm that leptospiral LPS activates mTLR4 and hTLR4, HB-mTLR4 and HB-hTLR4 cell lines were stimulated with serially diluted leptospiral LPS isolated from the whole-inactivated *Leptospira* preparations or *E. coli* LPS as positive control. Consistent with TLR4 activation by inactivated *Leptospira*, the measured responses induced by purified leptospiral LPS were higher in HB-mTLR4 compared to HB-hTLR4 cells. Purified leptospiral LPS from all four *Leptospira* strains activated mTLR4 dose-dependently and the level of mTLR4 activation was similar for all four LPS species (**Figure 4C** solid lines). In contrast, LPS isolated from serogroups Icterohaemorrhagiae and Canicola appeared as a significantly stronger activator of hTLR4 compared to the LPS isolated from serogroups Grippotyphosa and Bratislava (**Figure 4D** solid lines), suggesting that LPS isolated from different *Leptospira* strains may differentially activate hTLR4. Interestingly, a similar trend in the level of hTLR4 activation by leptospiral LPS, was observed in canine moDC (**Figure 2B**). Furthermore, polymyxin B treatment of purified leptospiral LPS completely inhibited the activation of hTLR4 by serogroups Canicola and Icterohaemorrhagiae (**Figure 4D** dotted lines). In contrast, although the activation of mTLR4 by *E. coli* LPS was completely abolished in the presence of polymyxin B, treatment with polymyxin B significantly reduced but not fully inhibited mTLR4 activation by LPS derived from serogroups Canicola and Grippotyphosa (**Figure 4C** dotted lines). However, mTLR4 activation by leptospiral LPS could be further inhibited when higher polymyxin B concentrations were used (**Supplementary Figure S6D**). These results suggest that leptospiral LPS investigated in this study can signal through TLR4 in both humans and mice.

### Whole-Inactivated *Leptospira* Activate Canine, Mouse and Human TLR2

**Figure 5:**
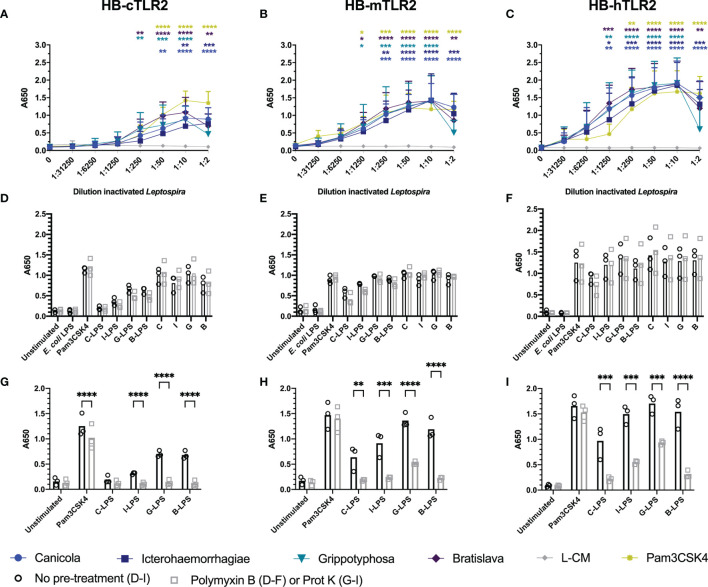
Inactivated *Leptospira* activate dog, mouse and human TLR2. HEK-Blue reporter cells expressing **(A)** dog (HB-cTLR2), **(B)** mouse (HB-mTLR2) and **(C)** human (HB-hTLR2) TLR2 were stimulated for 24 h with serially diluted Pam3CSK4 (yellow) or leptospiral culture medium (L-CM; grey) as positive and negative control, respectively or whole-inactivated *L. interrogans* serogroup Canicola (circle), Icterohaemorrhagiae (square) or Bratislava (diamond) or *L. kirschneri* serogroup Grippotyphosa (triangle). Pam3CSK4 was diluted 10-fold starting at 10 µg/ml. Mean values from three or four independent assays per cell line are shown. Each assay was performed using two or three replicates per condition. The error bars represent the SD. A mixed-effect analysis combined with Dunnett’s multiple comparisons test was used to test for significance between the stimulated cells and unstimulated control (0). **(D–F)** Cells were stimulated with *E. coli* LPS, Pam3CSK4 (controls), whole-inactivated *Leptospira* serogroup Canicola **(C)**, Icterohaemorrhagiae **(I)** or Bratislava **(B)** or *L. kirschneri* serogroup Grippotyphosa **(G)** or purified leptospiral LPS (C-LPS, I-LPS, G-LPS, B-LPS) either untreated (circle) or pre-treated with polymyxin B, at a final concentration of 50 µg/ml polymyxin B in cell culture (square). **(G-I)** Cells were stimulated with untreated (circle) or 1 mg/ml proteinase K pre-treated (square) Pam3CSK4 or purified leptospiral LPS (C-LPS, I-LPS, G-LPS, B-LPS). Mean values from three independent assays per cell line are shown **(D–I)**. Each assay was performed using three or four replicates per condition. A mixed-effect analysis combined with Šidák’s multiple comparisons test was used to test for statistical significance between the untreated and polymyxin B or proteinase K treated stimulus. No significant effect of polymyxin B was found. *p < 0.05, **p < 0.01, ***p < 0.001, ****p < 0.0001.

The canine TLR2 amino acid sequence cloned in the present study showed 76.8% identity and 88% similarity to human TLR2 (**Supplementary Figure S7A**), while it was 66.8% identical and 81.7% similar to mouse TLR2 (data not shown). The extracellular domains of canine and human TLR2 are visualized in **Supplementary Figure S7B** and demonstrate high similarity, suggesting similar ligand binding for both TLR2 species. To compare the activation of dog, mouse and human TLR2 by inactivated *Leptospira*, HB-cTLR2, HB-mTLR2 and HB-hTLR2 cells (**Figures 5A–C**) were stimulated with serially diluted Pam3CSK4 as a positive control and leptospiral culture medium as negative control, or inactivated bacteria of *L. interrogans* serogroup Canicola, Icterohaemorrhagiae, and Bratislava or *L. kirschneri* serogroup Grippotyphosa. Despite a cytotoxic effect at 1:2 dilution, all four inactivated *Leptospira* strains significantly activated canine, mouse and human TLR2-expressing reporter cells in a dose-dependent manner. In contrast to TLR4 activation, leptospiral culture medium did not induce TLR2 activation. Although the magnitude of TLR2 activation differed between dog, mouse and human, the level of activation induced by each *Leptospira* strain relative to each other was similar for all three TLR2 species, suggesting these four *Leptospira* strains induce similar TLR2 responses in dogs, mice, and humans (**Figures 5A-C**).

Literature indicates that leptospiral LPS signals through TLR2 in humans (27). To test this hypothesis in the canine setting, we stimulated canine TLR2 expressing cells with whole-inactivated *L. interrogans* serogroup Canicola, Icterohaemorrhagiae, and Bratislava or *L. kirschneri* serogroup Grippotyphosa, or their purified LPS, with and without polymyxin B treatment (**Figure 5D**). All four *Leptospira* strains activated canine TLR2. The fact that polymyxin B treatment did not reduce TLR2 activation by whole bacteria, indicated that canine TLR2 does not recognize leptospiral LPS. Furthermore, although purified leptospiral LPS activated canine TLR2, the activation was not affected by polymyxin B either, suggesting that TLR2 activation by these LPS preparations might be induced by contaminants such as lipoproteins. Interestingly, similar responses were observed in mouse and human TLR2 expressing reporter cell lines (**Figures 5E, F**), suggesting that TLR2 is not involved in the recognition of LPS derived from the *Leptospira* strains used in this study.

Even though the SDS-PAGE analysis of the purified leptospiral LPS preparations showed no major protein contamination (**Supplementary Figure S2B**), we expected TLR2 reporter cells to be more sensitive to lipoprotein contamination compared to SDS-PAGE. To confirm this, purified leptospiral LPS preparations were treated with proteinase K before stimulation of canine, mouse and human TLR2 expressing cells (**Figures 5G-I**). Proteinase K treatment had minor effect on Pam3CSK4 activation of TLR2 of all three species due to the absence of a proteinase K cleavage site in this synthetic lipopeptide. In contrast, proteinase K significantly reduced canine, mouse and human TLR2 activation by leptospiral LPS compared to the untreated LPS, suggesting that the observed activation of TLR2 was mainly driven by contaminating lipoproteins present in the LPS preparations. However, it should be noted that the proteinase K treatment did not inhibit mouse and human TLR2 activation by leptospiral LPS preparations completely (**Figures 5H, I** **)**. In addition, TLR2 and TLR4 expressing HEK-Blue cells were stimulated with leptospiral LPS pre-treated with different concentrations of polymyxin B (**Supplementary Figure S6**). While polymyxin B dose-dependently inhibited the activation of mTLR4 and hTLR4 by leptospiral LPS (**Supplementary Figure S6D, E**), the effect on the activation of cTLR2, mTLR2 and hTLR2 was negligible (**Supplementary Figure S6A-C**), supporting the hypothesis that TLR2 activation observed in this study was mostly due to the lipoproteins present in leptospiral LPS preparations.

### Heat-Treated, Whole-Inactivated *Leptospira* Activate Mouse and Human TLR5

**Figure 6:**
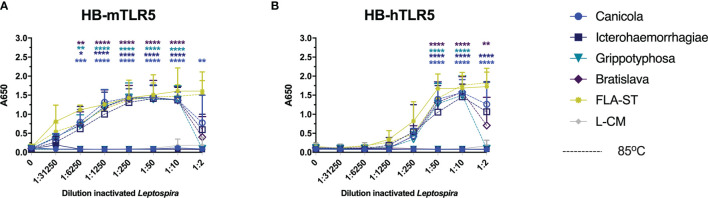
Heat-treated *Leptospira* activate TLR5. HEK-Blue reporter cells expressing **(A)** mouse (HB-mTLR5) and **(B)** human (HB-hTLR5) TLR5 were stimulated for 24 h with serially diluted FLA-ST (yellow) or leptospiral culture medium (L-CM; grey) as positive and negative controls, respectively or whole-inactivated *L. interrogans* serogroup Canicola (circle), Icterohaemorrhagiae (square) or Bratislava (diamond) or *L. kirschneri* serogroup Grippotyphosa (triangle), that were either heat-treated at 85°C (dotted lines with open symbols) or left untreated (solid lines with closed symbols). Mean values from three independent assays per cell line are shown. Each assay was performed using two or three replicates per condition. The error bars represent the SD. A mixed-effect analysis combined with Tukey’s multiple comparisons test was used to test for significance between the untreated and heat-treated stimulus at each dilution. *p < 0.05, **p < 0.01, ***p < 0.001, ****p < 0.0001.

Recently, it was discovered that the core leptospiral flagellar filament is covered by a shielding protein sheet, suggesting that intact *Leptospira* evade recognition by TLR5 (31). Another recent study showed that heat-treated *Leptospira* induced human TLR5 activation but not mouse TLR5 activation (32). To investigate whether the four leptospiral vaccine strains used in this study would behave similarly, HB-mTLR5 and HB-hTLR5 reporter cells were stimulated with serially diluted whole-inactivated *L. interrogans* serogroup Canicola, Icterohaemorrhagiae, and Bratislava or *L. kirschneri* serogroup Grippotyphosa that were either incubated at 85°C for 30 min or left untreated. In addition, cells were stimulated with serially diluted *Salmonella typhimurium* flagellin (FLA-ST) as positive control. Stimulation with FLA-ST induced a strong, dose-dependent activation of mTLR5 (**Figure 6A**) and hTLR5 (**Figure 6B**), while inactivated *Leptospira* did not activate these receptors. However, after heat treatment at 85°C, all four *Leptospira* strains induced a similar level of mouse and human TLR5 activation (**Figures 6A, B** **)**. Because we were not able to obtain a functional canine TLR5 reporter cell line, canine moDC were stimulated with heat-treated *Leptospira* instead. However, IL-1β mRNA expression was not enhanced by stimulation with heat-treated *Leptospira* (data not shown), indicating that this cell model is not suitable to study canine TLR5 involvement in detail. Although our findings confirm the recently reported observation that intact *Leptospira* escape TLR5 recognition, they do not support species-specific differences in TLR5 activation described for human and mouse TLR5 in the same study (32)

## Discussion

Leptospirosis, caused by Gram-negative *Leptospira*, is prevalent in dogs and bacterin vaccines against canine leptospirosis have been commercially available for decades (9). PRRs expressed by antigen-presenting cells interact with MAMPs present in the vaccines, and generate signals essential for the activation of the innate and ultimately the adaptive immune response (45, 46). According to literature, *Leptospira* induce TLR2 activation in mice and humans. In contrast, TLR4 activation is described in mice, a leptospirosis resistant species, but less clear in human cells (47). In addition, live intact *Leptospira* evade recognition by TLR5. However, when killed by antimicrobial peptides or heat-treated, leptospiral flagellin is exposed and recognized by human and bovine TLR5 (32). Because it is not known how *Leptospira* activate TLRs in dogs, we investigated whether canine TLR4 and TLR2 are involved in the recognition of four inactivated *Leptospira* strains, present in vaccines against canine leptospirosis. In addition, the response of human and mouse TLR2, TLR4 and TLR5 to the inactivated *Leptospira* vaccine strains was investigated to assess activation of these TLRs across different species.

Because reporter cell lines expressing canine TLRs were not readily available, we set up a canine moDC culture system to study *Leptospira*-induced innate immune responses in dogs. We first showed that canine moDC produce IL-1β, IL-6 and IL-12p40 in response to purified TLR ligands such as *E. coli* LPS, Pam3CSK4 and flagellin indicating that canine moDC express functional homologs of human and mouse TLR4, TLR2 and TLR5, respectively (**Figures 1A-C**). Next, by focusing on IL-1β production after stimulation with four inactivated *Leptospira* strains belonging to serogroups Canicola, Icterohaemorrhagiae, Grippotyphosa and Bratislava, we could show that all four strains induced IL-1β mRNA levels in canine moDC. Moreover, these responses were partially inhibited by the LPS-neutralizing reagent polymyxin B (**Figure 2A**). As whole-inactivated *Leptospira* contain multiple MAMPs, these results suggest that in addition to LPS other MAMPs such as lipoproteins are involved in the activation of canine moDC as well.

Experiments using the purified leptospiral LPS preparations alone or in combination with polymyxin B, confirmed that IL-1β upregulation following stimulation with *Leptospira* vaccine strains was LPS dependent, suggesting that canine TLR4 is involved in the recognition of leptospiral LPS in canine moDC (**Figures 2B, C** **)**. Activation of TLRs, including TLR2 and TLR4, by Gram-negative and Gram-positive bacteria has been shown to regulate bacterial internalization and phagosome maturation (48). We observed a reduction in the uptake of *Leptospira* in presence of polymyxin B and the TLR4 antagonist RS-LPS at 60 min. However, at 180 min the uptake of *Leptospira* in the presence of polymyxin B and RS-LPS had increased again (**Figure 3**). Results from another phagocytosis experiment showed similar outcome (**Supplementary Figure S4**). Since TLRs are not phagocytic entry receptors but only sensors, it is likely that next to other TLRs, receptors such as FcRs, complement and non-opsonic receptors expressed by phagocytes as well as production of pro-inflammatory cytokines and chemokines (49), enhance the bacterial uptake, thereby diminish the inhibitory effect of polymyxin B and RS-LPS. Nonetheless, the observation that polymyxin B and the RS-LPS reduced the uptake of fluorescently labeled *Leptospira* in canine moDC, suggests that *Leptospira* signals partially *via* TLR4 (**Figure 3**). Taken together, these results strongly suggest the presence of a functional TLR4 in canine moDC. In addition, these results support the hypothesis that next to TLR4 also other PRRs, such as TLR2 and TLR5, may be involved in the recognition of these *Leptospira* strains.

Since we were unable to clone and functionally express canine TLR4, together with canine MD2 and CD14 in HEK-Blue reporter cells, we used reporter cell lines expressing mouse and human TLR4, CD14 and MD2 to specifically investigate TLR4 responsiveness to four inactivated *Leptospira* vaccine strains. Our data show that both mouse and human TLR4-expressing reporter cells were activated by all four inactivated parental *Leptospira* strains and their LPS variants (**Figure 4**). These results are similar to the results obtained after stimulation of dog moDC with *Leptospira* or purified LPS (**Figures 2**, **3**) but contradict previous studies showing that leptospiral LPS is recognized predominantly by human TLR2 instead of TLR4 (27, 28). This discrepancy could be due to the different *Leptospira* strains used. To our knowledge, only *L. interrogans* serovar Icterohaemorrhagiae strain Verdun was previously used in human TLR activation studies (27, 28), the LPS of which may not be representative of all *Leptospira* spp. In fact, our data show that serogroup Grippotyphosa and Bratislava-derived LPS seem to be less potent human TLR4 activators compared to serogroup Canicola and Icterohaemorrhagiae (**Figures 4B, D** **)**, suggesting that human TLR4-activating capabilities by leptospiral LPS might be serovar dependent. In addition, it was recently reported that the inflammatory response to an infection with *L. interrogans* serovar Copenhageni strain Firocruz L1-130 in humanized TLR4/MD2 transgenic mice was comparable to the response of congenic wild-type mice expressing mouse TLR4, suggesting that functional human or mouse TLR4 is required to control infection by this strain (50). Alternatively, the differences between our observations and previously published results could also be explained by differences in the LPS purification method. In the present study, leptospiral LPS was purified from the aqueous phase of hot-phenol/water extraction which is in contrast to previous studies where leptospiral LPS from the phenol phase was used (27, 28). It has been described that the LPS from different *Leptospira* strains partitions in hot-phenol/water mixtures either preferably into the aqueous or the phenol phase (39, 51, 52). Taken together, our findings suggest that next to mouse TLR4, human TLR4 is also capable of recognizing leptospiral LPS and that this may depend on the leptospiral strain or serovar and whether leptospiral LPS was purified from the aqueous or phenol phase of hot phenol/water extraction.

In addition to TLR4, we also explored the involvement of canine TLR2 in the recognition of four *Leptospira* strains commonly used in vaccines against canine leptospirosis (**Figure 5**). As part of this study, HEK-Blue reporter cells expressing canine TLR2 were generated and used together with available HEK-Blue cell lines expressing human and mouse TLR2 to analyze TLR2 activation by the inactivated *Leptospira* strains across three species. It should be noted that HEK-Blue cell lines used, endogenously express human TLR1 and TLR6. All four inactivated *Leptospira* strains induced canine, human and mouse TLR2 activation in the reporter cells suggesting only minor variability in the TLR2-activating properties of the four *Leptospira* strains tested. The measured response of canine TLR2 activation in transfected cells was lower compared to mouse and human TLR2. However, it remains unclear whether this observation is a result of canine TLR2 being intrinsically less sensitive to *Leptospira* compared to human and mouse TLR2 or whether this is caused by technical differences between the reporter cells. Thus, a direct comparison of canine, human and mouse TLR2 responses with each other cannot be made (**Figures 5A-C**). Nevertheless, our results are in line with previous studies demonstrating human and mouse TLR2 activation by *Leptospira* (53, 54).

In addition to the classical activation of TLR2 by lipoproteins, Werts et al. (2001) reported that LPS isolated from an *in vitro* passaged *L. interrogans* strain is recognized by human TLR2 and that TLR2 activation could be inhibited by addition of polymyxin B (27). We investigated whether LPS isolated from the inactivated *L. interrogans* serogroup Canicola, Icterohaemorrhagiae, and Bratislava or *L. kirschneri* serogroup Grippotyphosa could activate TLR2 in our system as well. Although leptospiral LPS preparations activated dog, mouse and human TLR2, the activation was not inhibited by addition of polymyxin B (**Figures 5D–F** and **Supplementary Figure S6A–C**), which is in contrast to the findings reported by Werts *et al.* (27). This suggests that the observed activation of TLR2, was not induced by the leptospiral LPS. Importantly, proteinase K treatment of the purified leptospiral LPS used in this study significantly reduced canine, mouse and human TLR2 activation, suggesting that lipoproteins were co-purified with the leptospiral LPS preparations (**Figures 5G–I**). We did, however, not further investigate which lipoproteins are activating TLR2 specifically. Additional studies with ultrapure *Leptospira* LPS are needed to confirm whether the residual TLR2 activation by leptospiral LPS that was detected after proteinase K treatment in our study could be LPS dependent or due to remaining lipoproteins inaccessible to degradation with proteinase K.

Although *Leptospira* spp. are flagellar bacteria, it is less clear how these bacteria induce innate immune activation through TLR5. A recently published study demonstrated that only after exposure of *Leptospira* to antimicrobials or heat treatment, these bacteria activated human and bovine but not mouse TLR5 (32). We cloned and expressed canine TLR5 containing a FLAG tag in HEK-Blue reporter cells. Although we confirmed the expression of FLAG by spot blot, we were not able to confirm the functionality of canine TLR5 after stimulation with inactivated *Leptospira* or *Salmonella typhimurium* flagellin (data not shown). As an alternative, we used reporter cell lines expressing mouse or human TLR5 (**Figure 6**). Our findings show that intact inactivated *L. interrogans* serogroup Canicola, Icterohaemorrhagiae, and Bratislava or *L. kirschneri* serogroup Grippotyphosa do not activate mouse or human TLR5. Only after heat-treatment of *Leptospira*, both mouse and human TLR5 were activated by these bacteria. These results partially align with a previous study (32), however, in our hands, all four heat-treated *Leptospira* strains activated both mouse and human TLR5 similarly. In addition, heat treatment of inactivated *Leptospira* did not enhance IL-1β production in canine moDC compared to intact inactivated bacteria (data not shown). Although our data show that canine moDC express a functional TLR5 by recognizing FLA-ST (**Figure 1A**), IL-1β production may not be sensitive enough to detect moDC activation by leptospiral flagellin specifically due to simultaneous activation of other TLRs by leptospiral MAMPs such as LPS. Additional studies are needed to unravel this.

In summary, our findings show that TLR4 and TLR2 are involved in the recognition of whole-inactivated *Leptospira* in dogs, humans, and mice. Moreover, we demonstrated that canine moDC as well as human and mouse TLR4-expressing cell lines recognize purified leptospiral LPS and that, in contrast to other studies, these LPS variants do not seem to activate TLR2 of these species. Furthermore, evasion of TLR5 recognition by inactivated, but intact *Leptospira* suggests that leptospiral flagellin and TLR5 signaling do not contribute to the induction of immune responses against *Leptospira*. Consequently, it seems likely that both TLR2 and TLR4 are necessary for the induction of innate immune responses against inactivated *L. interrogans* serogroup Canicola, Icterohaemorrhagiae, and Bratislava or *L. kirschneri* serogroup Grippotyphosa strains *in vivo* and hence against canine *Leptospira* vaccines consisting of these same serovars. Previously it has been shown that TLR2 mRNA was upregulated in canine whole blood stimulated with *Leptospira* (55). However, our study provides evidence of functional TLR2 and TLR4 responses to *Leptospira*-derived MAMPs in dogs. Together, our results shed light on the activation of dog TLRs by canine *Leptospira* vaccine antigens and contribute to more detailed understanding of TLR activation by *Leptospira*.

## Data Availability Statement

The original contributions presented in the study are included in the article/**Supplementary Material**. Further inquiries can be directed to the corresponding author.

## Author Contributions

AN, FB, and AS designed the research. AN performed the research. EP performed MS analysis. EV performed confocal microscopy. AN analyzed the data. FB and AS supervised the work. AN, EP, VR, FB, and AS wrote the paper. All authors contributed to the article and approved the submitted version.

## Funding

This project has received funding from the Innovative Medicines Initiative 2 Joint Undertaking under grant agreement no. 115924 (VAC2VAC). This joint undertaking receives support from the European Union’s Horizon 2020 research and innovation program and EFPIA.

## Author Disclaimer

The contents of this article represent the authors’ scientific conclusions and neither IMI nor the European Union, EFPIA, or any Associated Partners are responsible for any use that may be made of the information contained therein.

## Conflict of Interest

Authors EP and AS were employed by company Intravacc. Author AN was affiliated with company Intravacc at the time of study but was not formally employed.

The remaining authors declare that the research was conducted in the absence of any commercial or financial relationships that could be construed as a potential conflict of interest.

## Publisher’s Note

All claims expressed in this article are solely those of the authors and do not necessarily represent those of their affiliated organizations, or those of the publisher, the editors and the reviewers. Any product that may be evaluated in this article, or claim that may be made by its manufacturer, is not guaranteed or endorsed by the publisher.
