# Supplementary material for: Activation of Canine, Mouse and Human TLR2 and TLR4 by Inactivated Leptospira Vaccine Strains
Source: Front Immunol. 2022 Mar 21;13:823058. doi: 10.3389/fimmu.2022.823058 (PMC8978998; doi:10.3389/fimmu.2022.823058)
Supplement: Supplementary file 1 [file DataSheet_1.pdf]

Supplementary figures

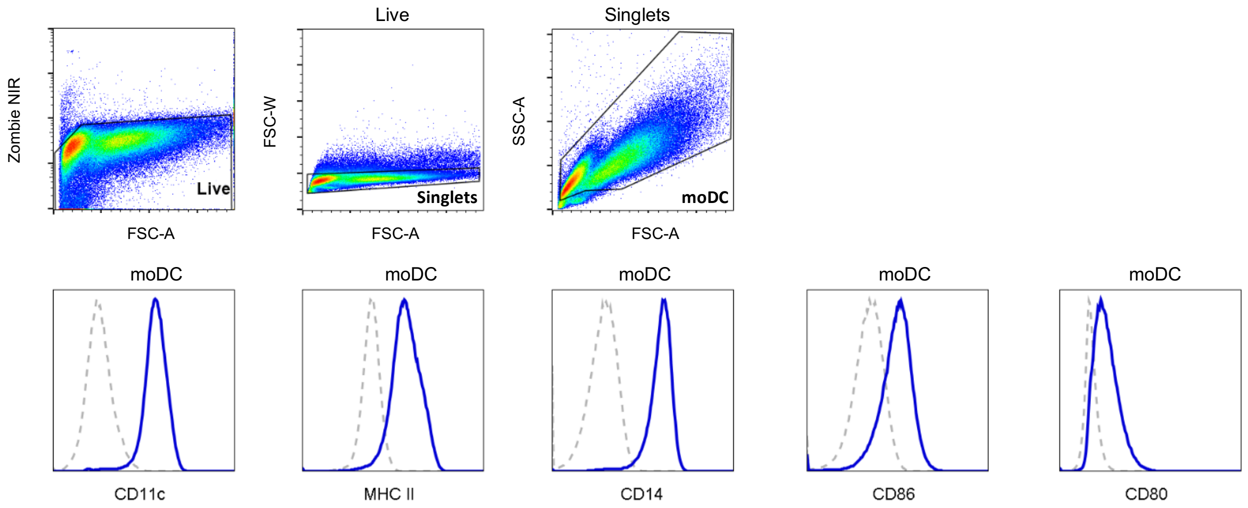

Supplementary Figure S1. Flow cytometric analysis of canine moDC differentiated from dog monocytes. MoDC were differentiated for 6 days and analyzed by flow cytometry. Cells were gated on live, single cells before analyzing the expression of CD11c, MHC II, CD14, CD80 and CD86 surface markers in the moDC population. Fully stained (blue) and FMO controls (grey) are overlaid on the histograms. Representative plots are shown. FSC-A- forward scatter area, FSC-W- forward scatter width, FMO-fluorescence minus one control.

(A) (B)

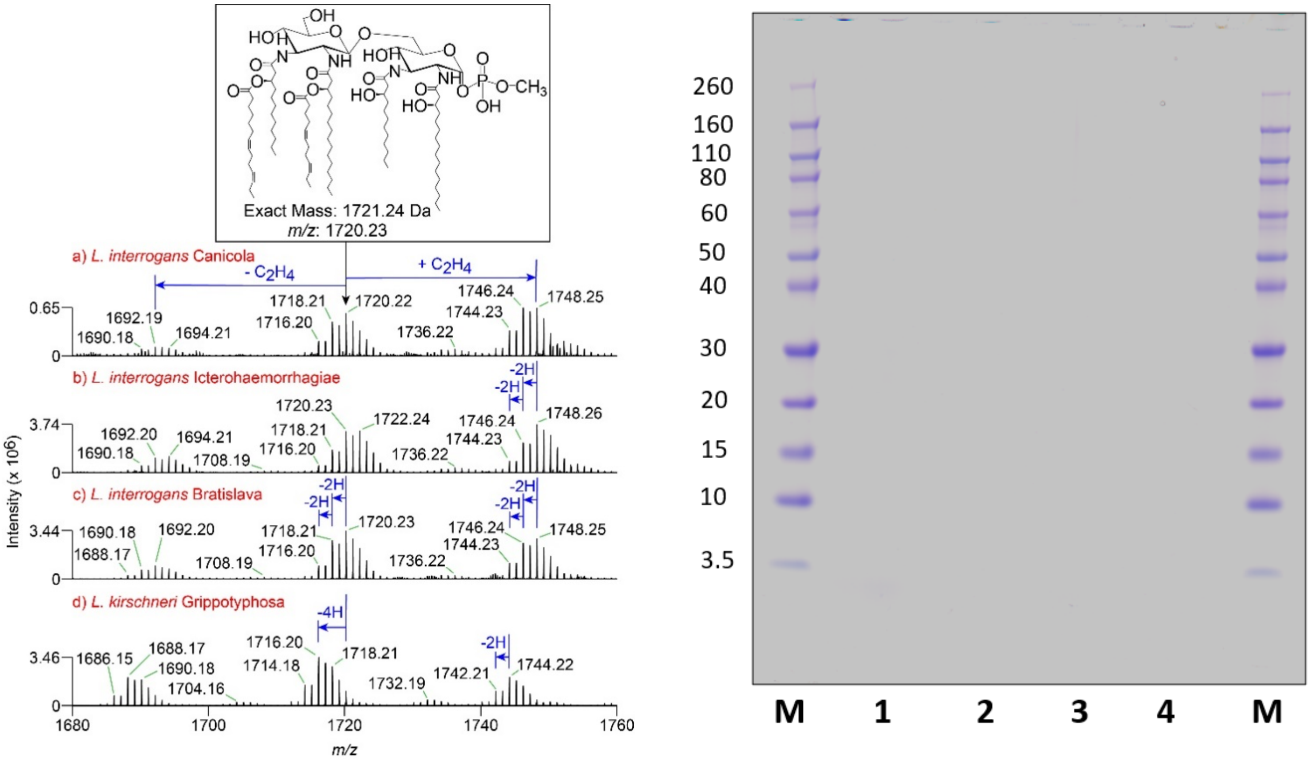

Supplementary Figure S2. Analysis of isolated leptospiral LPS preparations. (**A**) MS analysis of the leptospiral lipid A. The sections of average mass spectra encompassing singly deprotonated lipid A ions were generated by in-source CID nLC-FT-ESI-MS of the LPS isolated from *L. interrogans* serogroup Canicola (a), Icterohaemorrhagiae (b) and Bratislava (c) and *L. kirschneri* serogroup Grippotyphosa (d). Mass spectra were obtained by averaging single MS scans over the whole elution time span of LPS molecules. *m/z* values correspond to monoisotopic peaks. The structure assigned to the ion peak of *m/z* 1720**.**23 is shown on top of the spectra (44). The location of carbon-carbon double bonds in fatty acids of leptospiral lipid A is not known (47). (**B**) Five µl of LPS preparations isolated from inactivated serogroups Icterohaemorrhagiae (lane 1), Canicola (lane 2), Grippotyphosa (lane 3) and Bratislava (lane 4) were applied to 4-12% Bis-Tris SDS-PAGE and stained with Coomassie. Lane M: Pre-stained protein standard molecular weight marker.

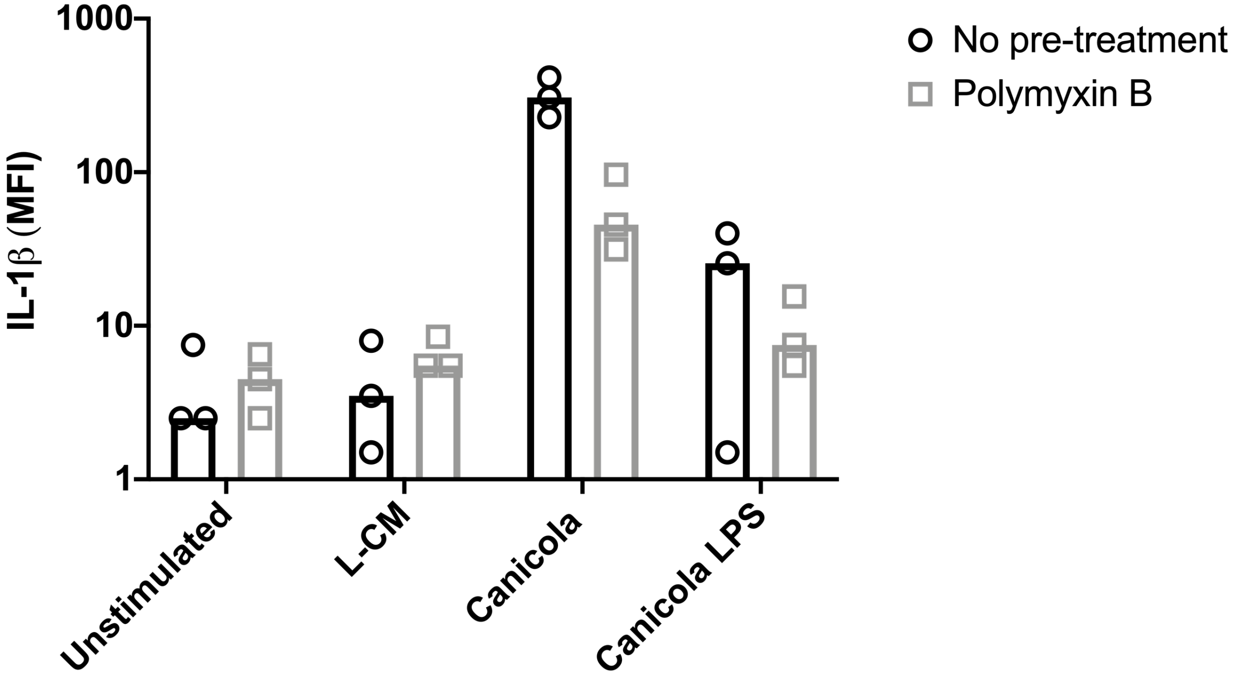

Supplementary Figure S3. IL-1β production following stimulation with inactivated *Leptospira* or leptospiral LPS. IL-1β protein expression was measured in the supernatants of canine moDC stimulated with leptospiral culture medium as control, whole inactivated serogroup Canicola or its purified LPS for 48 h (circle). In addition, moDC were stimulated with the same stimuli which were pre-treated with polymyxin B (square). Data from one experiment performed in three replicates per stimulation is shown. MFI-median fluorescence intensity.

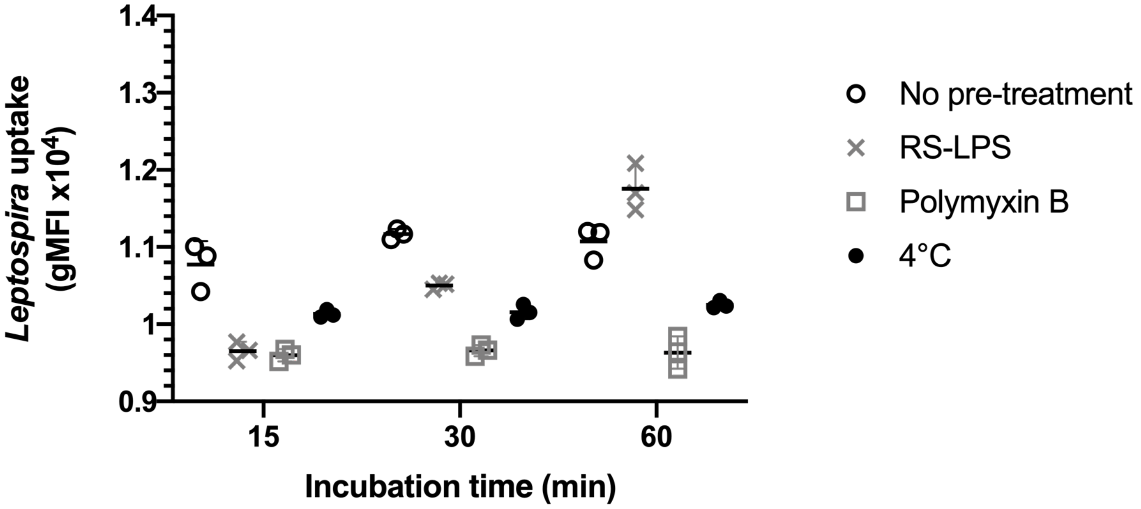

Supplementary Figure S4. The uptake of inactivated Leptospira in canine moDC. Canine moDC were incubated with FITC-labeled inactivated *Leptospira* for 15, 30 or 60 min at 37ºC (open circle) or 4ºC (closed circle). In addition, moDC were pre-treated with RS-LPS (cross) or stimulated with *Leptospira* that were pre-incubated with polymyxin B (square). The experiment was performed in triplicate for each condition and *Leptospira* uptake was determined by flow cytometry as an increase in FITC geometric mean fluorescence intensity (gMFI) in the MHC II positive gate.

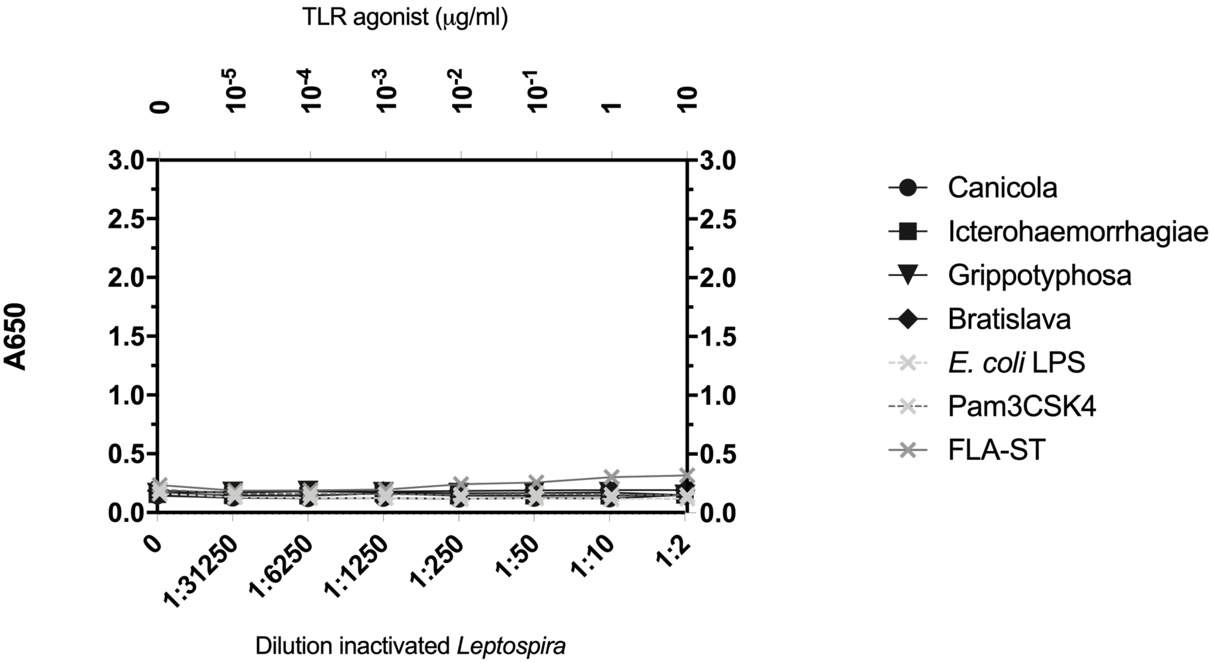

Supplementary Figure S5. Stimulation of parental cell line HEK-Blue-Null1 with TLR agonists or whole-inactivated *Leptospira* does not induce SEAP production. HEK-Blue-Null1 reporter cells were stimulated for 24 h with serially diluted *E. coli* LPS (light grey dotted line), Pam3CSK4 (dark grey dotted line), FLA-ST (dark grey solid line) or whole-inactivated *L. interrogans* serogroup Canicola (circle), Icterohaemorrhagiae (square) or Bratislava (diamond) or *L. kirschneri* serogroup Grippotyphosa (triangle). Stimulations were performed in duplicate, and the error bars represent the SD.

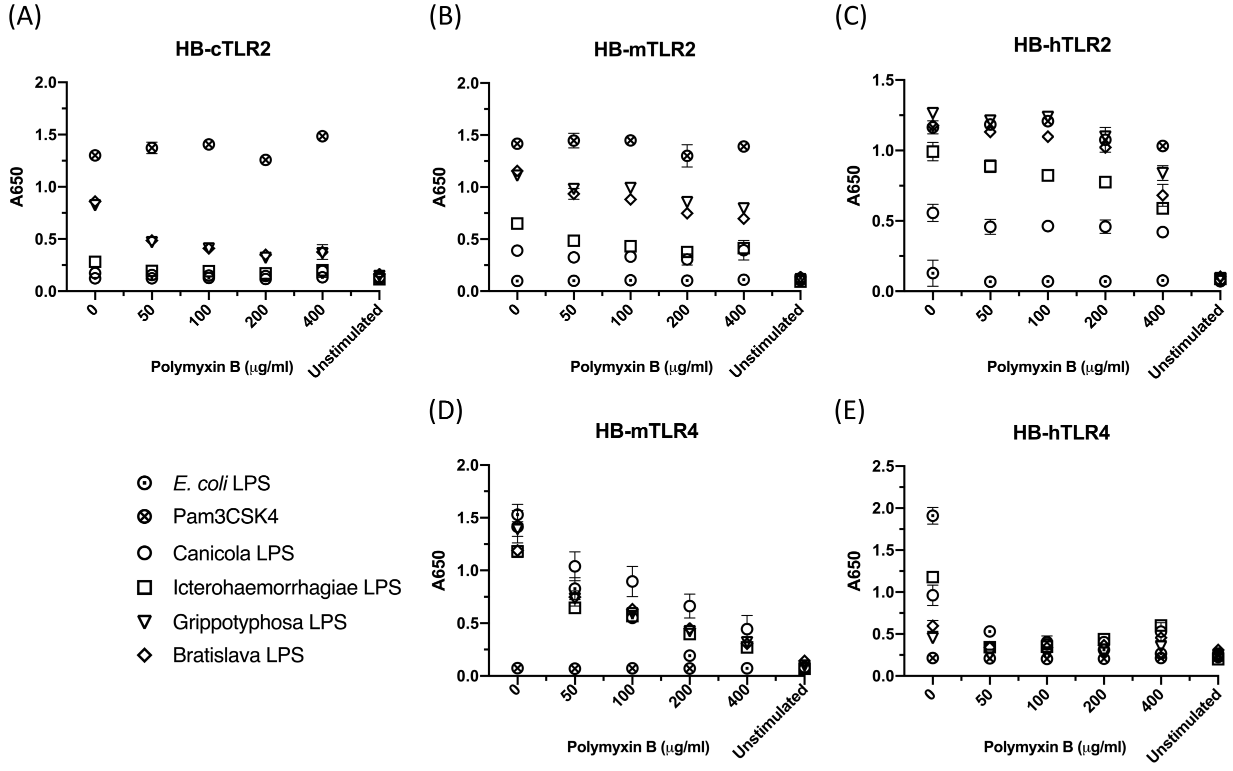

Supplementary Figure S6. Polymyxin B does not inhibit TLR2 activation by leptospiral LPS. HEK-Blue reporter cells were stimulated with *E. coli* LPS (dot circle), Pam3CSK4 (crossed circle) or purified LPS from *L. interrogans* serogroup Canicola (circle), Icterohaemorrhagiae (square) or Bratislava (diamond) or *L. kirschneri* serogroup Grippotyphosa (triangle) in presence of different concentrations of polymyxin B. Activation of (**A**) dog (HB-cTLR2), (**B**) mouse (HB-mTLR2) and (**C**) human (HB-hTLR2) TLR2-expressing reporter cells is shown, as well as activation of (**D**) mouse (HB-mTLR4) and (**E**) human (HB-hTLR4) TLR4-expressing reporter cell lines is shown. Stimulations were performed in duplicate and data from a representative experiment are shown. The error bars represent the SD.

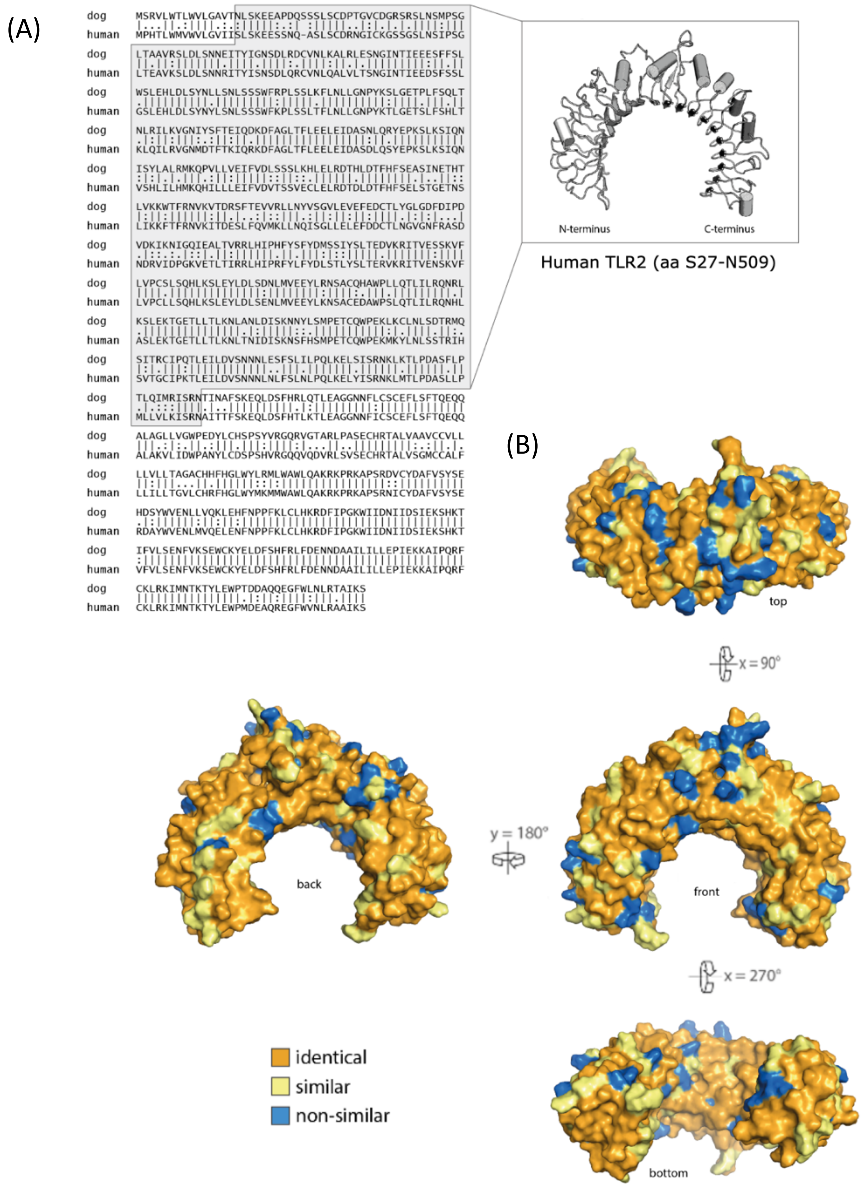

Supplementary Figure S7. Alignment of dog and human TLR2 amino acid sequences and structural similarities between predicted extracellular domains. (**A**) Pairwise sequence alignment of canine and human TLR2 was performed with EMBOSS Needle (https://www.ebi.ac.uk/Tools/psa/emboss_needle/). Identical (line), similar (two dots) and non-similar (one dot) amino acids are shown. The sequence of the predicted extracellular domain is indicated in the shaded box. The crystal structure of the human TLR2 extracellular domain (PDB identifier 6NIG) (top right) was used as a basis to generate (**B**) a homology model of canine and human TLR2 extracellular domain in different orientations. Identical (orange), similar (yellow) and non-similar (blue) amino acid residues in canine and human TLR2 were visualized using PyMOL software.
